# Effects of Gyejibongnyeong-hwan on dysmenorrhea caused by blood stagnation: study protocol for a randomized controlled trial

**DOI:** 10.1186/1745-6215-13-3

**Published:** 2012-01-05

**Authors:** Jeong-Su Park, Sunju Park, Chun-Hoo Cheon, Ho-Yeon Go, Seung-Ho Sun, Yong-Cheol Shin, Bo-Hyoung Jang, Seong-Gyu Ko

**Affiliations:** 1 Center for Clinical Research and Genomics, Oriental Medical College, Kyung Hee University, Seoul, Republic of Korea; 2 Department of Preventive Medicine, Oriental Medical College, Kyung Hee University, Seoul, Republic of Korea; 3 Department of Oriental Internal Medicine, Korean Medicine College, Semyung University, Chungju, Republic of Korea; 4 Department of Oriental Internal Medicine, Korean Medicine College, Sangji University, Wonju, Republic of Korea

## Abstract

**Background:**

Gyejibongnyeong-hwan (GJBNH) is one of the most popular Korean medicine formulas for menstrual pain of dysmenorrhea. The concept of blood stagnation in Korean medicine is considered the main factor of causing abdominal pain, or cramps, during menstrual periods. To treat the symptoms, GJBNH is used to fluidify the stagnated blood and induce the blood flow to be smooth, reducing pain as the result. The purpose of this trial is to identify the efficacy of GJBNH in dysmenorrhea caused by blood stagnation.

**Methods:**

This study is a multi-centre, randomised, double-blind, controlled trial with two parallel arms: the group taking GJBNH and the group taking placebo. 100 patients (women from age 18 to 35) will be enrolled to the trial. Through randomization 50 patients will be in experiment arm, and the other 50 patients will be in control arm. At the second visit (baseline), all participants who were already screened that they fulfil both the inclusion and the exclusion criteria will be randomised into two groups. Each group will take the intervention three times per day during two menstrual cycles. After the treatment for two cycles, each patient will be followed up during their 3^rd^, 4^th ^and 5^th ^menstrual cycles. From the screening (Visit 1) through the second follow-up (Visit 6) the entire process will take 25 weeks.

**Discussion:**

This trial will provide evidence for the effectiveness of GJBNH in treating periodical pain due to dysmenorrhea that is caused by blood stagnation. The primary outcome between the two groups will be measured by changes in the Visual Analogue Score (VAS) of pain. The secondary outcome will be measured by the Blood Stagnation Scale, the Short-form McGill questionnaire and the COX menstrual symptom scale. Analysis of covariance (ANCOVA) and repeated measured ANOVA will be used to analyze the data analysis.

**Trial registration:**

Current Controlled Trials: ISRCTN30426947

## Background

Dysmenorrhea is a very common medical condition in women worldwide. The prevalence varies from 45% to 95% of all women depending on its definition [1]. In Korea, 78.3% of all adolescent girls have dysmenorrhea during their menstrual periods [2]. The most common symptom of dysmenorrhea is abdominal pain, or cramp. It is not only painful but interferes with daily activities, and it has been the leading cause of recurrent short-term school absence [3].

The most prominent physiological cause of dysmenorrhea in allopathic medicine is the production of uterine prostaglandins. Endometrial cells release prostaglandins when they are deciduous. The released prostaglandins stimulate myometrial contractions and cause ischemia. This results in abdominal pain, which has been the source of difficulties that women have to cope with in their daily lives.

But the explanation for the pain is different in Traditional Korean Medicine. In Traditional Korean Medicine, the causes of sicknesses are classified according to symptoms and signs. Traditional Korean Medical specialists diagnose the cause of the sickness by symptoms and signs and prescribe the appropriate remedy.

The most common factor causing dysmenorrhea in Traditional Korean Medicine is blood stagnation in the uterus. 'Flow' is a very important concept in Traditional Korean Medicine. If the flow of *qi *and blood inside the both is smooth, the body is healthy without any diseases. But if the flow is interrupted, it causes pain. This can be applicable to *qi *and blood. Blood stagnation means obstructed blood flow, and a woman experiencing dysmenorrhea can be explained by blood stagnation in her body. The signs of blood stagnation are getting bruised easily, tender abdominal pain, etc.

The first treatment of dysmenorrhea in allopathic medicine is an over-the-counter drug, especially non-steroidal anti-inflammatory drugs (NSAIDs) such as Ibuprofen, Naproxen, Mefenamic, etc. [3,4]. These drugs, however, have failed to show effect in 20% to 25% of women, and moreover some women complain about digestive disorders while taking the drug. In this case, Korean herbal medicine can be a plausible alternative [5].

Gyejibongnyeong-hwan (GJBNH) is one of the most popular Korean medicine formulas for periodical pain due to dysmenorrhea. GJBNH fluidifies blood to induce smooth blood flow and reduce pain [6]. However, the evidence of the effect of GJBNH in treating dysmenorrea has been reported mostly in the form of case reports. In the era of evidence medicine, high quality evidence is mandatory for a medical formula to be proven effective.

The purpose of this trial is to identify the efficacy of GJBNH in dysmenorrhea caused by blood stagnation.

## Methods

### Objectives & Hypothesis

#### Objectives

1) Clinically significant improvement in the severity of pain caused by dysmenorrhea

2) Correlation between blood stagnation level and pain severity

#### Hypothesis

The hypothesis is that GJBNH will reduce menstrual pain more effectively than placebo after taking the intervention--GJBNH or the placebo--for two menstrual cycles.

### Setting

**Figure 1:**
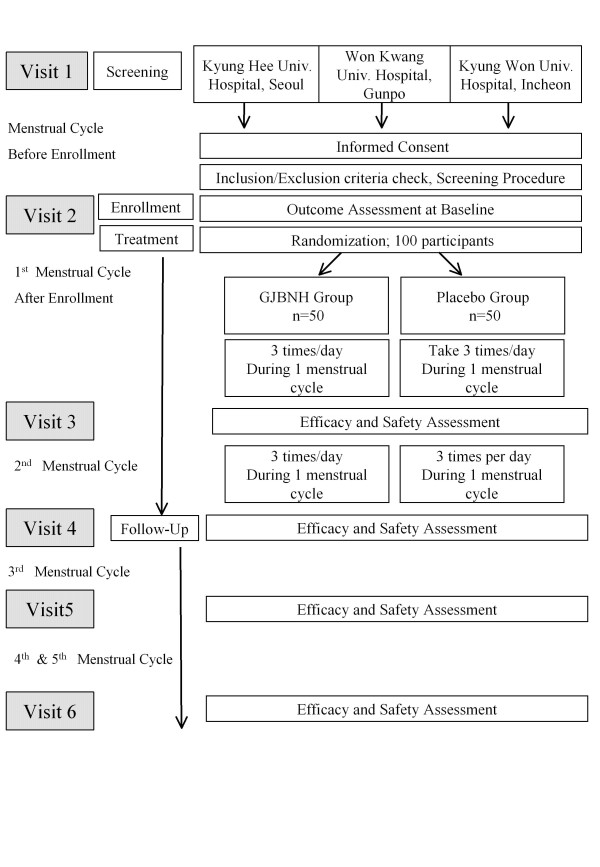
**Study flow chart**.

This trial is a randomized, double-blind, parallel group, placebo-controlled phase IV trial. There are three investigational sites: Oriental Medicine Obstetrics and Gynecology Clinic of Kyung Hee Medical Center in Seoul, Obstetrics and Gynecology Clinic of Won Kwang Oriental Hospital in Gunpo, and Obstetrics and Gynecology Clinic of Kyung Won Gil Oriental Medical Hospital of Kyung Won University in Incheon, Korea. Participants will be treated as outpatients in these sites. Before the beginning of the trial, the protocol of the trial has been approved by the Institutional Review Board (IRB). All participants will take GJBNH or placebo for two menstrual cycles (about eight weeks). They will be followed up during three menstrual cycles. It takes five menstrual cycles (about 20 weeks) from baseline to finish. The total span from screening (visit 1) to finish (2^nd ^follow-up) can vary according to the participant's period. Figure 1 is an overview of this trial.

### Participants

#### Inclusion Criteria

The inclusion criteria are as follow (Table 1).

**Table 1:** Inclusion criteria of GJBNH study

**Inclusion criteria**

1. Female aged 18 to 35 years.
2. Women whose period cycle was 30 ± 3 days during last 3 months.
3. Women who have menstrual pain (dysmenorrhea) over 6 degrees by the Visual Analogue Scale (VAS) at screening.
4. Women who are diagnosed with blood stagnation by two oriental medical gynecologic specialists.
5. Have given a written informed consent form
6. Have given a written informed consent form for genetic study.

#### Exclusion Criteria

The exclusion criteria of this trial are described in Table 2.

**Table 2:** Exclusion criteria of GJBNH study

**Exclusion criteria**

1. A woman who has major neuro-psychiatric disorder (schizophrenia, epilepsy, alcohol abuse, anorexia, etc.) or has history of major neuro-psychiatric disorder.
2. A woman who is planning to have a baby.
3. A woman who is taking anti-depressant, anti-serotonin, barbiturate, or psychotropic drugs.

### Interventions

Gyejibongnyeong-hwan (GJBNH) is a Traditional Korean Medicine formula. It consists of *Cinnamomi ramulus, Poria, Moutan cortex, Persicae semen*, and *Paeoniae radix*. Gyejibongnyeong-hwan is a small brown ball-like tablet. A single dose of GJBNH is one pouch in which there are 20~30 tablets. The placebo medicine is made of lactose, corn starch and food coloring and has a similar appearance, shape, weight, taste, and color as GJBNH. The treatment drug and placebo will be provided by Hanpoong Pharm & Foods Co., Ltd.

### Rescue medication

Ten pain killer pills will be given during each treatment cycle as rescue medication. If the participant can not endure the pain, she can take the rescue medication. But all participants should not take any pain-killer except given by the investigator. If the participant takes any other drug than what is given from the trial, it should be described in the case-report form (CRF). Any participant taking drugs that can influence the outcome such as NSAIDs or oral contraceptive pills will be excluded from the trial.

### Outcomes

#### Primary outcome

The primary outcome in this study is the change in the Visual Analogue Scale (VAS) of average menstrual pain between the baseline (Visit 2) and after the treatment (Visit 4). A 100 mm VAS will be used to assess average level of pain during the menstrual period.

#### Secondary outcome

The secondary outcome measures include the VAS (the maximum pain during the menstrual period), the Blood Stagnation Scale, the Short Form McGill Pain Questionnaire [7], and the Cox Menstrual Symptom Scale [8]. The quantity of pain killer pills taken during the menstrual period would be recorded at every visit. Heart Rate Variability (HRV) will also be measured.

### Safety assessment

All participants will report any adverse event she had while taking the intervention at every visit. Every adverse event will be described in the CRF. If the adverse event is severe and associates with the trial, the participant will be withdrawn from the trial and appropriate treatment will be given to her. For safety assessment, a liver function test, blood cell count test, physical examination, and urine analysis will be carried out at Visit 4.

### Sample size

The primary outcome in this trial is the change of the Visual Analogue Score (VAS) of average pain in the menstrual period between the baseline (Visit 2) and after treatment (Visit 4). The hypothesis is

H0:δ=Δ1-Δ2=0

H1:δ=Δ1-Δ2≠0

Δ1: The change of VAS score between Visit 4 and baseline in GJBNH group

Δ2: The change of VAS score between Visit 4 and baseline in placebo control

As a reference, we have chosen the study of Choi et. al. [6] and Yeh LL [9]. The mean (± standard deviation, SD) change of the VAS in GJBNH group (n = 13) was 2.42 (± 2.04) and those in placebo control group (n = 38) was 1.08 (± 2.14). We used the mean difference between groups, 1.34 (= 2.42-1.08), as a clinically significant improvement worth to detect and 2.12 as its pooled standard deviation. With 5% of two-sided significance level and 80% of statistical power, we needed to randomly assign fifty participants in each group considering approximately 20% of drop-out rate.

### Randomization

Participants will be given a random number at Visit 2. When a participant makes the second visit to the trial site, the investigator will connect to the Medical Research Collaboration Center (MRCC) web site run by Seoul National University Hospital which is commissioned for the allocation process in this trial. To randomize, the investigator will connect to the MRCC website (http://mrcc.snuh.org) and input patient's number and birthday, and confirm that the patient fulfil the inclusion and the exclusion criteria. Then, a random number is generated. No one except the researcher at the MRCC can know whether the number indicates treatment group or placebo group, and all participants, investigators, and monitors are blinded.

### Statistical method

Efficacy analysis will be performed for both ITT (intention-to treat, all randomly assigned participants) and PP (per-protocol, participants completed the trial without any protocol deviations) data sets. To compare the differences of the average changes of the VAS between the experimental arm and the control arm, the Short Form McGill Pain Questionnaire score, the blood stagnation score, the Cox menstrual score from the baseline (Visit 2) to after the treatment (Visit 4) between two groups, and analysis of covariance (ANCOVA) using the baseline score as a covariate will be used.

For the variables which measure the autonomic nerve system (HF, high frequency; LF, low frequency; HF/LF ratio), similar methods described above or their corresponding non-parametric analysis will be performed as appropriate.

For ITT analysis, missing data will be imputed by MI (Multiple Imputation) method using SAS/MI procedure.

The safety data set will include the participants who take the intervention at least once. For safety analysis, physical examination and self-reported adverse event will be compared by the chi-square test. In addition, the results of liver function tests, blood cell count and urine analysis will be analyzed using the same methods as described in efficacy analysis after classifying these results into normal or abnormal according to their respective normal ranges. The chi-square test will be used to compare the differences between the groups.

### Monitoring

The Center for Clinical Research and Genomics (CCRG; the CRO) is responsible for monitoring. Monitoring will begin after the first participant completes the whole process of this study. Every institution where the trial is being conducted will be monitored while this trial is in process using the Standard Operation Procedure (SOP)s.

### Ethical Consideration

The trial is conducted to the Declaration of Helsinki 2008 and/or the regulations of the "Good Clinical Practice" principles in the Korea Food & Drug Administration.

The Institutional Review Board (IRB) has approved of this clinical trial at all institution before the participant recruitment. The reference numbers are KOMC IRB 2008-07 (IRB of Kyung Hee Oriental Medical Center, approved on 18^th ^of Aug 2008), WONSBHB IRB 2009-02 (IRB of Wonkwang University Sanbon Oriental Medical Center, approved on 24^th ^of Feb 2009) and 09-101 (IRB of Kyungwon Gil Oriental Medical Hospital approved on 2^nd ^of Feb 2009). Prior to undertaking any study-related procedures, all participants will provide written informed consent.

## Discussion

It is not a facile task to design a trial to identify the efficacy of Traditional Korean herbal formula, because the sickness has to be diagnosed according to various symptoms and signs, and in Traditional Korean Medicine herbal formula has to be decided according to different diagnosis. The symptoms and signs are critical, not the name of the disease--this led the inclusion and exclusion criteria to be as diverse and thorough as the subjects of the trial.

Dysmenorrhea has typical symptoms and characteristics and thus can be categorized into types based on certain symptoms. The types vary according to diseases, and in this case dysmenorrhea can be defined to four types of causes: blood stagnation, coldness in the uterus, blood and *qi *deficiency and weak constitution of liver and kidney [10]. The remedies vary according to the cause, but blood stagnation is the most assessable among these types while the other types are difficult to evaluate. This is why we have selected blood stagnation as the cause of dysmenorrhea that we treat. There are many Traditional Korean Medicine formula treating blood stagnation to cure dysmenorrhea, but GJBNH is the most common and widely used with rare adverse events.

Determining the sample size is another challenge. There are few references, so we used two reference papers to calculate the sample size.

Gyejibongnyeong-hwan as one of Traditional Korean Medicine formula has been widely used for thousands of years. But, the evidence supporting this formula is rare. We are hoping that this trial will provide high quality evidence and that the protocol for this trial will be a reference in design further clinical trials.

## Competing interests

The authors declare that they have no competing interests.

## Authors' contributions

JSP, SJP, CHC and BHJ have written the first manuscript for this trial and calculated the sample size. They will monitor this trial. YCS, HYG, SHS and SGK have edited the first manuscript. SGK has conducted all the procedures for this protocol. All authors read and approved the final manuscript. 

**Table 3:** Summary of assessments at procedures

	Screening	Treatment period			Follow-up	
	
	Visit 1	Visit 2	Visit 3	Visit 4	Visit 5	Visit 6
Informed Consent	x					

History Taking^1^	x					

Demographic information taking^2^	x					

Pregnancy Check^3^		x	x	x	x	x

Blood Stagnation Diagnosis^4^	x	x	x	x		

VAS^5^	x	x	x	x	x	x

McGill Questionnaire	x	x	x	x	x	x

Cox Menstrual Scale	x	x	x	x	x	x

Physical Examination	x			x		

Liver function test^6^	x			x		

Blood Cell Count^7^	x			x		

Urinalysis^8^	x			x		

Safety Assessment^9^			x	x	x	x

HRV	x	x	x	x		

Compliance			x	x		

• Visit 1; Informed consent and screening procedure

•Visit 2; Randomization and outcome assessment for baseline

•Visit 4; Outcome assessment for primary objective

x: Item has to be carried out for the visit

^1 ^history of drug taking for the past one month, allergy and disease history, obstetric history, menarche and menstrual period, etc.

^2 ^date of birth, age, height, and weight.

^3 ^medical examination by interview.

^4 ^a scale for diagnosing blood stagnation, has not been published.

^5 ^the 100 mm visual analogue scale. The mean and the maximum pain during the menstrual period

^6 ^AST, ALT, BUN, creatinine, ALP

^7 ^WBC, RBC, hemoglobin, hematocrit, Platelet

^8 ^protein, glucose, urobilinogen, ketone, RBC, WBC squamous cell

^9 ^medical examination and self-report of adverse event
